# Predicting the decision making chemicals used for bacterial growth

**DOI:** 10.1038/s41598-019-43587-8

**Published:** 2019-05-10

**Authors:** Kazuha Ashino, Kenta Sugano, Toshiyuki Amagasa, Bei-Wen Ying

**Affiliations:** 1 Graduate School of Life and Environmental Sciences, University of Tsukuba, Ibaraki, 305-8572 Japan; 2 Graduate School of Systems and Information Engineering, University of Tsukuba, Ibaraki, 305-8573 Japan; 3 Center for Computational Sciences, University of Tsukuba, Ibaraki, 305-8577 Japan

**Keywords:** Machine learning, Applied microbiology, Systems analysis

## Abstract

Predicting the contribution of media components to bacterial growth was first initiated by introducing machine learning to high-throughput growth assays. A total of 1336 temporal growth records corresponding to 225 different media, which were composed of 13 chemical components, were generated. The growth rate and saturated density of each growth curve were automatically calculated with the newly developed data processing program. To identify the decision making factors related to growth among the 13 chemicals, big datasets linking the growth parameters to the chemical combinations were subjected to decision tree learning. The results showed that the only carbon source, glucose, determined bacterial growth, but it was not the first priority. Instead, the top decision making chemicals in relation to the growth rate and saturated density were ammonium and ferric ions, respectively. Three chemical components (NH_4_^+^, Mg^2+^ and glucose) commonly appeared in the decision trees of the growth rate and saturated density, but they exhibited different mechanisms. The concentration ranges for fast growth and high density were overlapped for glucose but distinguished for NH_4_^+^ and Mg^2+^. The results suggested that these chemicals were crucial in determining the growth speed and growth maximum in either a universal use or a trade-off manner. This differentiation might reflect the diversity in the resource allocation mechanisms for growth priority depending on the environmental restrictions. This study provides a representative example for clarifying the contribution of the environment to population dynamics through an innovative viewpoint of employing modern data science within traditional microbiology to obtain novel findings.

## Introduction

Bacterial growth is one of the most representative phenomena of living systems^1,2^, thus, it is foundational in microbiology and the topic has been touched on in countless ways. Nevertheless, the decision factors involved in bacterial growth remain unclear because growing cells are highly dynamic rather than stagnant networks. In addition to the genome-wide information that provides static perspectives of cells^3–6^, intensive studies from the perspective of cell population dynamics have addressed decision factors by introducing computational simulation and mathematic modelling^7–9^. Biologically well-known regulatory mechanisms are often investigated to construct reliable theoretical models using experimental measurements^10,11^. These studies successfully clarified the cellular activity or population growth of designated genetic structures in response to a defined genetic or environmental stimulus^12^. However, bacterial growth is decided by countless factors (*e*.*g*., biomolecules and chemicals) of either intrinsic or extrinsic origin, and even a simple bacterial cell shows a multi-dimensional, complicated landscape^13^. Thus, growth is somehow too complex to explain using defined equations with a few manually selected parameters. Considering that millions of nonlinear biochemical reactions occur simultaneously in a living cell, it is unfeasible to include all kinetic parameters in a writable model. Lately, the metabolism flux balance analysis (FBA), which requires no kinetic details, was developed^14^ and successfully applied to understand the growing bacteria at the steady state^15,16^. As these models are currently constructed under the limited conditions and do not take the ions into account, whether the growth could be predicted from a broad range of chemical landscape is under investigation.

The current machine learning technique could be a practical approach. Given a large experimental data set, it can find implicit relationship between explanatory and objective variables only from the data itself without any biological mechanisms. Machine learning is being successfully applied in life sciences, mostly in the fields of imaging technology^17–20^, genetic analyses and sequence prediction^21–26^. To date, these studies have mostly addressed questions about the genetic reasons for fitness changes^27–29^ as well as our efforts to explore growth-correlated changes in the transcriptome, genome and mutations of *E*. *coli*^30–32^. Since living cells experience through their lives under a given chemical condition, the environmental contribution not only to an organism’s omics^33,34^ but also to its fitness must be addressed. In studying bacterial growth as a data science, growth prediction could be conducted by including a large number of patterns in input information, such as the culture conditions, without the details of the complex reaction or regulation mechanisms.

The contribution of the external conditions, in particular, the media, to bacterial growth have been analysed through direct experimentation to a great extent^35–37^. To obtain hints about the decision making factors involved in bacterial growth, the major substrates essential for bacterial growth (*e*.*g*., glucose) were extensively studied^38,39^. However, the variety of the substrate compositions was still limited. The chemical concentrations were usually controlled within a reasonable range of one or two orders, and the combinations of the compositions were often around dozens. Thus, a high-throughput experimental survey on the bacterial growth in a broad range of chemical concentrations and a large number of combinations are required for the test by means of machine learning.

In the present study, whether the population growth of the asexual populations could be explained by the chemical components is examined, by the high-throughput growth assay associated with the machine learning for the first time. The present study is intended to address the contribution of the chemical components in the media to key indexes of bacterial growth, i.e., the growth rate and the saturated density^40–42^. These parameters represent the population fitness response to the environment and the environmental capacity for the population, respectively. Precise and high-throughput growth assays were performed to acquire sufficient experimental records of bacterial growth for data analysis. To obtain an alternative view of bacterial growth and ultimately extract insight from the growth data, decision tree learning^43,44^, which is one of the multifaceted disciplines in data science^45^, was introduced. This approach could lead to meaningful decisions as to where to mark the boundaries among the ranges of attributed chemicals to split the different branches of a growth parameter tree. This process continues, with branches being recursively split into smaller, more specific branches for different attributes (chemicals), with the tree leaves being classes. This study successfully predicted the growth decision chemicals and provided intriguing findings about the working mechanisms of the growth decision priority of these chemicals. These findings could be considered in the synthetic construction and metabolic design of living systems.

## Results

### Growth data acquisition under hundreds of chemical combinations

**Figure 1:**
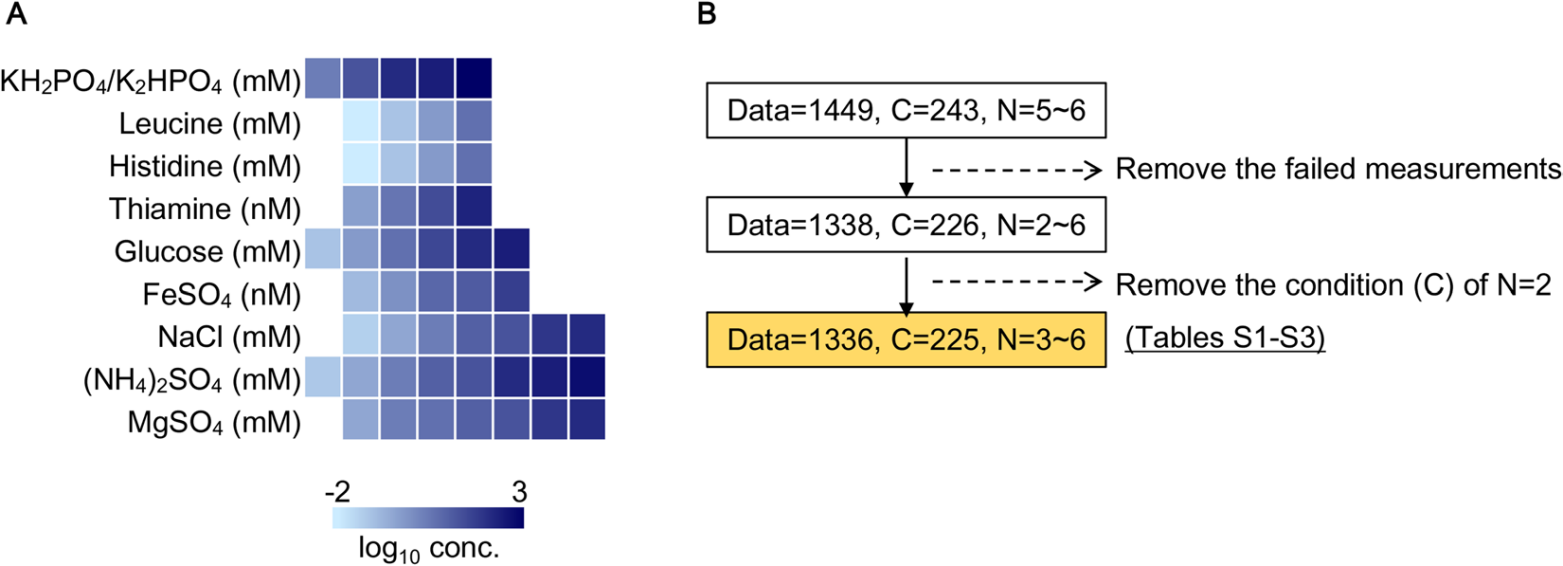
Overview of the experimental conditions and the data set. (**A**) Chemical compounds used in the growth assay. The heat map represents the variation in the concentrations of the chemical compounds used in the experiments. The gradation from light to dark blue indicates the concentration from low to high, over a logarithmic scale. (**B**) Flowchart of the data refinement. Data, C, and N represent the numbers of the individual growth curves acquired in the assay, the tested combinations made up of the ten chemical compounds, and the biological replicates per combination, respectively. The data set highlighted in orange was primarily analysed to reach the conclusion.

The *E*. *coli* strain MDS42^46^ was used for the growth analysis by means of machine learning, and the basic chemical compounds for bacterial growth were used, including the essential resources carbon and ammonium and the necessary metal ions, vitamins and amino acids (Fig. 1A). These chemical components were chosen according to the compositions of commonly used and/or previously reported synthetic media^31,47^. The chemical components that commonly appear in these media were selected as the essential components (*e*.*g*., glucose, PO_4_^+^), and those that only appeared in some of the synthetic media were chosen as accessory components (*e*.*g*., Na^+^, histidine). The base concentrations of these chemical components were referred to as synthetic media M63, M9 and MAA^31,47^, and they were altered independently. To acquire a growth landscape that was as broad as possible, the chemical concentrations were altered in order (Fig. 1A), and they were largely out of the ranges generally used in laboratories. In addition, to maintain the buffering effect (pH) of the culture conditions, the ratio of K_2_HPO_4_/KH_2_PO_4_ remained constant in all the combinations (Fig. 1A).

In particular, high-throughput growth assays of varied chemical compositions were performed with a 96-well microplate format, as carefully described elsewhere^48^. In brief, a total of 243 chemical combinations (C = 243) were tested repeatedly (N = 5~6), resulting in 1449 growth curves (Fig. 1B). After removing the unreliable measurements (i.e., the incomplete growth curves), a total of 1336 growth curves (Tables S2 and S3) representing 225 chemical combinations (Table S1) were subjected to growth analysis. Subsequently, a new program for the automated calculation of the exponential growth rate (*r*) and the saturated population density (*K*) from the growth curves (microplate measurements) was developed (Figs S1–S3, details in the Materials and Methods) with Python^49^. This program provided an accurate data processing platform (Fig. S1) for acquiring a reliable growth data set, which linked the chemical composition to the growth parameters, i.e., the growth rate and saturated density. Note that the further refinement of the data set by removing the irregular data did not change the conclusion of the present study (Figs. S6–S7, details in the Discussion).

### Overview of the growth and chemical combination data sets

**Figure 2:**
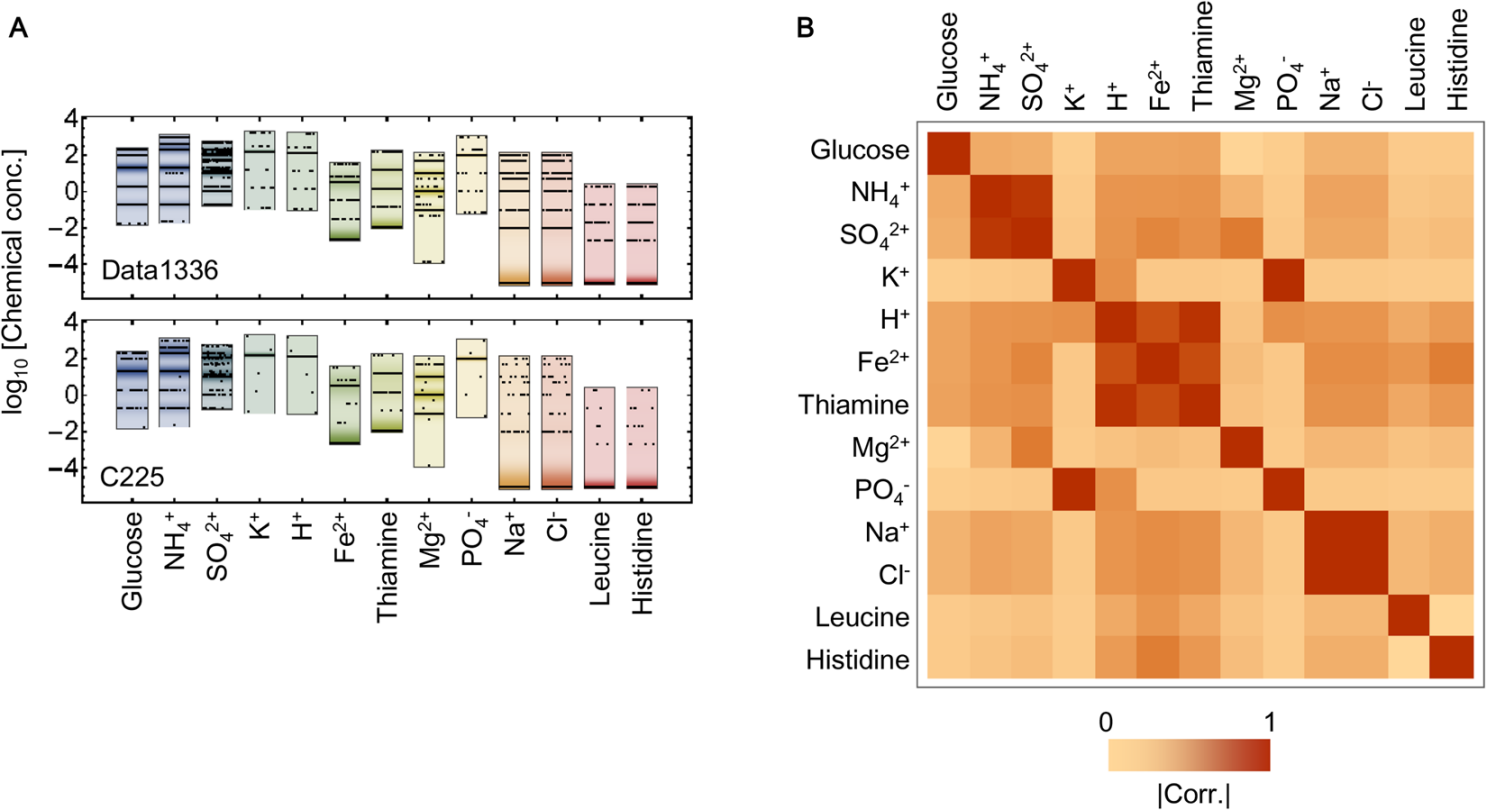
Data distribution and chemical combinations. (**A**) Data distribution at the single chemical level. A total of 13 chemicals, which are encompassed within the ten compounds, are indicated. The data distributions at the individual chemical levels are shown in coloured bars. The tested concentrations of these 13 chemicals are spotted in black, within the corresponding distribution bars. Those condensed spots appeared as black lines. The upper and bottom panels indicate the distribution of 1336 individual growth curves and the 225 combinations, respectively. (**B**) Relationships in the concentration changes of 13 individual chemicals. The matrix represents the correlations of the changes to the concentrations of any two chemicals. The gradation from light to dark orange indicates the correlation coefficients (Corr.) from low to high. The chemical elements found in the same compounds usually show high correlations, *e*.*g*., the chemical elements of Na^+^ and Cl^−^, which form the NaCl compound.

Considering that the chemical compounds (Fig. 1A) were soluble in water, the evaluation was performed on their ions rather than the chemical compounds initially used in the experiments. The transformation resulted in 13 variates, and the concentrations of these variates (chemical components) were transformed into logarithmic values for the analysis (Fig. 2A) because their concentrations were changed in order. Although the random changes in concentrations of all the chemical components were favoured to achieve an unbiased data set (landscape), it was impractical to avoid the correlated changes completely, of which the chemical components appeared in the same compounds, such as K^+^ and PO_4_^+^, NH_4_^+^ and SO_4_^2−^, Na^+^ and Cl^−^ (Fig. 2B, dark orange). Nevertheless, the overall change patterns showed that the concentrations of most chemical components were altered independently (Fig. 2B, light orange).

**Figure 3:**
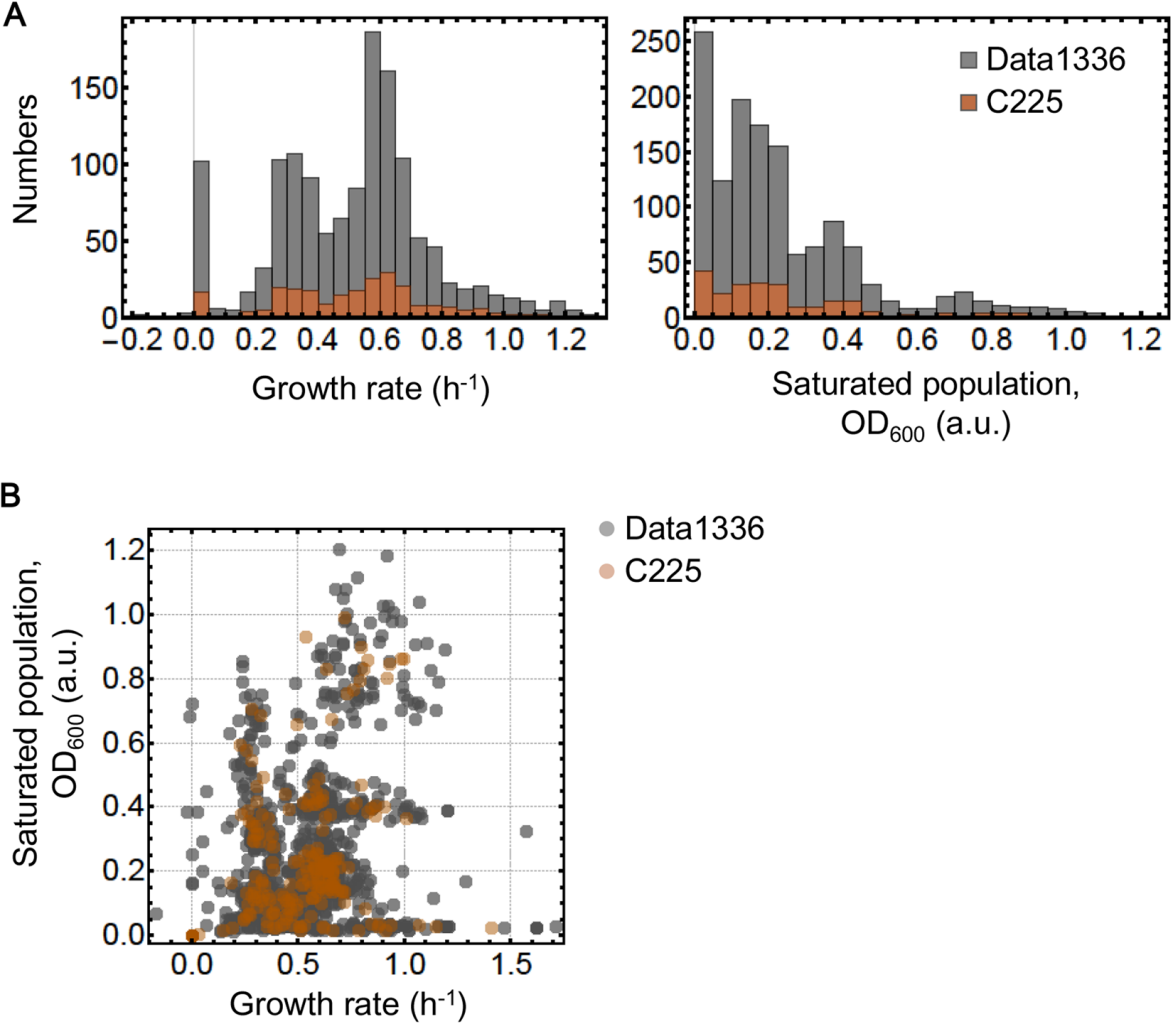
Calculated growth rates and saturated population density. (**A**) Histograms of the growth rate and the saturated density. The left and right panels show the histograms of the growth rate and the saturated density, respectively. (**B**) Relation between the growth rate and the saturated density. The Spearman rank correlation coefficients of Data1336 and C225 are 0.32 (*p* = 8e-34) and 0.54 (*p* = 1e-18), respectively. The data sets of Data1336 and C225 are indicated in grey and bronzed, respectively.

Both the growth rates of 1336 individual growth curves (Data1336, grey) and the mean growth rates under 225 chemical combinations (C225, brown) presented bimodal distributions when excluding zero growth (Fig. 3A, left). Multi-peak distributions of saturated density were commonly observed in both the individual population and the combination average (Fig. 3A, right). Based on these results, it was difficult to manually identify the key chemical components that played a decisive role in the growth rate and the saturated density. In addition, a weak but significant positive correlation (*p* < 0.001) between the growth rate and the saturated density (Fig. 3B) implied that the chemical combinations that accelerated the population growth also increased the population size. However, it was unclear whether the correlated changes were attributed to the same chemical components. To predict the key chemical components that determined the growth rate and/or the saturated density of high priority, decision tree learning^43^ was applied. Because the statistics of the data sets based on the individuals and combinations were equivalent (Fig. 3), the conclusions drawn from the individual growth data (Data1336) were primarily described.

### Prediction of the growth decision chemicals

**Figure 4:**
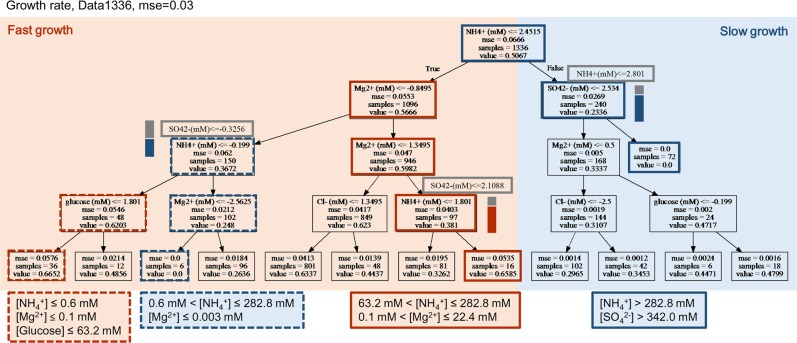
Decision tree of the growth rate. The data set of Data1336 was applied to the decision tree learning to predict the growth rate. The resulting tree with a maximal depth of four is shown, and its accuracy is indicated with cross validation (mse). The chemicals predicted to be the decision elements for the growth rate appear in the tree. Orange and blue illustrate the branches of fast and slow growth, respectively. The name of the selected chemical and its concentration in a logarithmic scale for bifurcation, the value of the cross validation of this selection, the number of data used for this selection, and the mean growth rate of the data used for this selection are summarized in the squares, from top to bottom. The squares involved in the paths of the best and worst chemical combinations for growth are highlighted with bold lines in orange and blue, respectively. The boxes represent the alternative chemicals predicted with the decision tree. The dual-colour bars beside the boxes indicate the frequencies of the alternatives at the same levels, revealing the stability of the tree/prediction. The chemical combinations predicted to cause either fast or zero growth are summarized in the large squares, in which the ranges of the chemical concentrations are shown at the linear scales.

**Figure 5:**
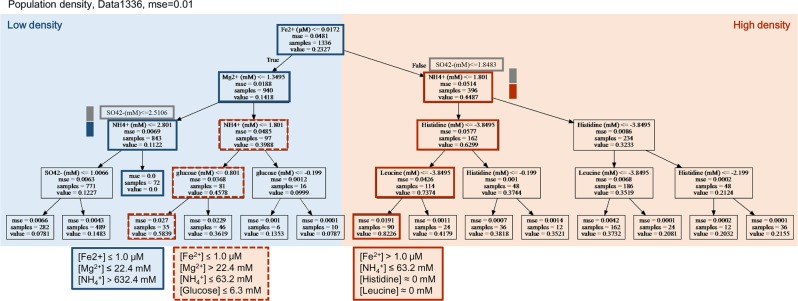
Decision tree of the saturated density. The data set for Data1336 was applied to decision tree learning to predict the saturated population density. The resulting tree with a maximal depth of four is shown, and its accuracy is indicated with cross validation (mse). The chemicals predicted to be the decision elements for the saturated density appear in the tree. Orange and blue illustrate the branches of high and low density, respectively. The name of the selected chemical and its concentration in a logarithmic scale for bifurcation, the value of cross validation for this selection, the number of data used for this selection, and the mean saturated density of the data used for this selection are summarized in the squares, from top to bottom. The squares involved in the paths of the best and worst chemical combinations for population density are highlighted with bold lines in orange and blue, respectively. The boxes represent the alternative chemicals predicted with the decision tree. The dual-colour bars beside the boxes indicate the frequencies of the alternatives at the same levels, revealing the stability of the tree/prediction. The chemical combinations predicted to cause either high or zero density are summarized in the large squares, in which the ranges of the chemical concentrations are shown in the linear scales.

To predict the growth decision makers among the 13 chemical components, a non-parametric decision tree learning, classification and regression tree (CART)^45,50^ was used. As a first trial for introducing machine learning into bacterial growth analysis, four-level decision trees were generated (Figs. 4 and 5), which were cross validated (mse <0.05) The decision maker that was the limiting factor in the growth rate was predicted to be the ammonium ion (NH_4_^+^) (Fig. 4). This finding indicated that the changes in the NH_4_^+^ concentration affected the growth rate most significantly, despite the changes in the combinations of the other 12 components. In addition, four chemical components, Mg^2+^, SO_4_^2−^, Cl^−^ and glucose, appeared in the decision tree, which could be roughly divided into two branches of fast and slow growth (Fig. 4, in orange and blue, respectively). It seemed that NH_4_^+^ and Mg^2+^directed both the growth (orange lines) and death paths (blue lines). For example, for the best growth performance, the NH_4_^+^ and Mg^2+^ concentrations were required to be from 63.2 to 282, and from 0.1 to 22.4 mM, respectively (orange solid lines). Either an excess amount of NH_4_^+^ (blue solid lines) or the depletion of Mg^2+^ (blue broken lines) would cause zero growth. Intriguingly, glucose played a decisive role when both NH_4_^+^ and Mg^2+^ were out of the optimal ranges (orange broken lines). This finding indicated that it was the nitrogen source but not the carbon source that presented the highest priority in deciding the growth rate. It well agreed with the previous finding of that glucose was no longer the best carbon source in the nitrogen poor conditions^38^.

However, the top decision maker for saturated density was the ferric ion (Fe^2+^), although both NH_4_^+^ and Mg^2+^ participated in the decision paths directing the largest or zero population sizes (Fig. 5). Nevertheless, the high density could be achieved in the condition of either high or low concentration of Fe^2+^, depending on Mg^2+^ and glucose (orange boxes at the bottom). It indicated that the high concentrations of both Fe^2+^ and Mg^2+^ could reduce the population size, probably due to the metal toxicity^36,51^. Interestingly, the two amino acids histidine and leucine were not required to achieve a high density (orange solid lines). The decision making factor for the population density was NH_4_^+^ but not the amino acids in the poor nutritional condition possibly due to the costly gene expression, as more proteins were required for the amino acids uptake. Taken together, out of the 13 chemical components, the two ions NH_4_^+^ and Fe^2+^ played determining roles in the growth rate and the saturated density, respectively. Although the weak positive correlation between the growth rate and the saturated density was observed (Fig. 3B), the optimal compositions of culture media differed in terms of the growth speed and growth maximum, which somehow represented the functional differentiation of the chemical elements of life.

Additionally, the primary nodes (NH_4_^+^ and Fe^2+^, respectively) of the decision trees were identical between cross-validation replicates. The exception was that SO_4_^2−^ appeared statistically instead of NH_4_^+^ (Figs 4 and 5, grey boxes and bars), which was because the NH_4_^+^ and SO_4_^2−^ appeared in the same (NH_4_)_2_SO_4_ compound and changed in a highly correlated manner (Fig. 2B). Since the trees were relatively stable, the decision tree was a proper method, to understand the present data sets and the analytical observations were reliable.

### Diverse mechanisms of the growth decision chemicals

**Figure 6:**
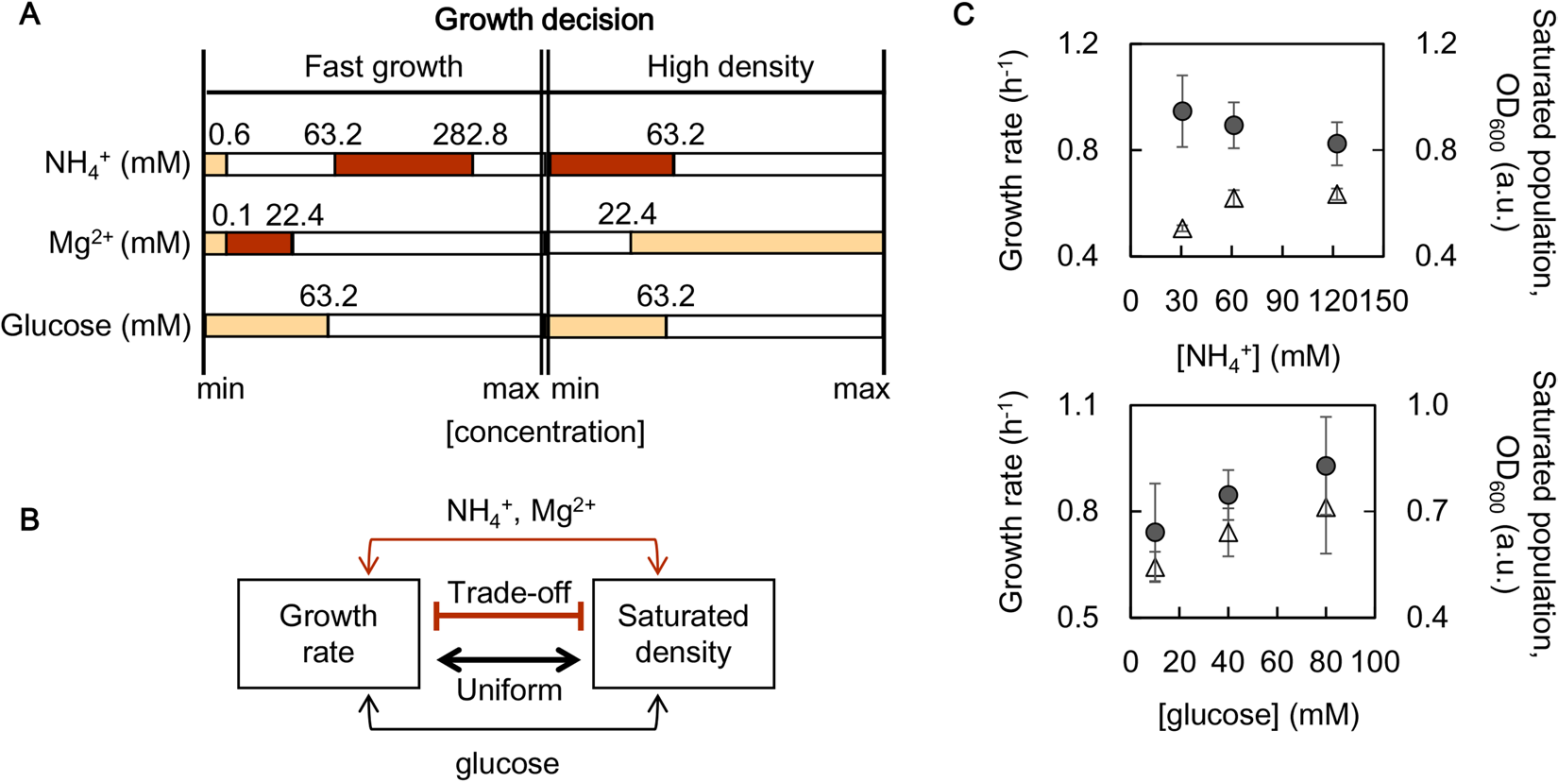
Mechanisms of the decision chemicals. (**A**) Growth priority decided by the chemical concentrations. The three most common decision chemicals are indicated. The predicted ranges of chemical concentrations optimal for either fast growth or high density are illustrated in the coloured bars. The concentrations of these elements at the branching points are indicated. Two varied choices of chemical combinations are indicated in light and dark orange, respectively. (**B**) Scheme showing the trade-off and uniform modes. The NH^4+^ and Mg^2+^ elements worked in a trade-off manner (bold orange) and glucose worked in a uniform manner (bold black) in determining the growth priority of the speed or quantity. (**C**) Experimental verification of the trade-off and the uniform contributions of the decision elements. The growth rates and the saturated densities at varied NH^4+^ and glucose concentrations (upper and lower panels, respectively) are represented by open triangles and filled circles, respectively. The standard errors of repeated tests (N = 5~6) are indicated.

According to the decision tree learning, three chemical components, i.e., NH_4_^+^, Mg^2+^, and glucose, were predicted to be common decision makers for both the growth rate and the saturated density (Figs. 4 and 5). The predicted optimal concentrations for either fast growth or high density were identical in glucose, but they differed for NH_4_^+^ and Mg^2+^ (Fig. 6A). To improve the growth speed and the maximum, the preferred glucose concentrations were lower than 63.2 mM in common. Comparatively, the optimal concentrations of NH_4_^+^ for fast growth were 63.2~282.8 mM, which were out of the optimal concentration range (<63.2 mM) for the high density. Similarly, the optimal concentrations of Mg^2+^ were lower and higher than 22.4 mM for fast growth and high density, respectively. Thus, glucose, the only carbon source in the present study, determined the growth speed and maximum in a uniform manner; however, the ammonium and magnesium ions (NH_4_^+^ and Mg^2+^) worked in a trade-off fashion (Fig. 6B). Note that these intriguing findings could not be achieved when applying the common mathematic analysis, such as, multivariate regression, which was failed in determining the chemicals correlated to the population density and observed three chemicals contributing to the growth rate (Table S4).

To verify the different mechanisms newly observed by decision tree learning, an experimental examination of NH_4_^+^ and glucose were performed. The base chemical composition was determined according to the minimal medium M63, in which the NH_4_^+^ and glucose concentrations were approximately 63.2 and 22.4 mM, respectively. The increased concentration of NH_4_^+^ (i.e., (NH_4_)_2_SO_4_) was accompanied by an increasing growth rate and a decreasing saturated density (Fig. 6C, upper). This finding clearly demonstrated that NH_4_^+^ determined the growth in a trade-off manner. By contrast, changes in the glucose concentration led to changes in the growth rate and the saturated density in the same direction (Fig. 6C, bottom). This finding supported the prediction of the overlapping concentration range for both fast growth and high density (Fig. 6A). The experiments confirmed the distinctive mechanisms of these chemical components of growth decisions. The trade-off and uniform working styles revealed the diversity in the utilization and allocation mechanisms of the chemical components participating in cellular life processes.

## Discussion

For the first trial to introduce machine learning into the growth analysis and prediction, whether and how much the purity or quality of the data sets effected the prediction (data mining) were evaluated. We found that using the data set of the averaged growth values of each combination (C225) led to the same conclusion (Figs. S4 and S5) as that of the individual growth values (Figs. 4 and 5). The highly identical prediction consequences revealed highly reduced errors over the repeated experiments and calculations. Reduced errors resulted from well-established experimental manipulation^48^ and the improved calculation program (Figs. S1–S3). Additionally, whether the highly purified data sets (Data1208, C213) would alter the conclusion was evaluated (Fig. S6). The distributions of the growth rate and the saturated density acquired from the highly purified data sets (Fig. S7) were highly comparable with those acquired from the noisy data sets (Fig. 3). Note that SO_4_^2−^ and H^+^ appeared statistically instead of NH_4_^+^ and thiamine, respectively (Figs. S8 and S9), due to the correlated changes in their concentrations (Fig. 2B). Overall, the major decision paths for the fast growth and high density predicted by these processed data sets (Figs. S8 and S9) were equivalent to those of the noisy data sets (Figs. 4 and 5), although an alternative path for high density was also formed (Figs. S8 and S9, highlighted in green). Since there was no significant alteration in the predicted results, to avoid biased or over-learning with the cleaned-up data sets, using noisy data sets for machine learning was appropriate.

The most intriguing finding of the prediction was that the ammonium ions (NH_4_^+^) served as the top-level decision maker for bacterial growth and determined the growth speed and maximum in a trade-off manner. The nitrogen in the ammonium ions was an essential chemical element for the biosynthesis of nucleotides, the building blocks of the DNA and RNA genetic molecules. The nucleotide (purine and pyrimidine) metabolism was directly associated with the amino acid metabolism^52,53^, in which nitrogen took part. The nitrogen-mediated mechanism probably ran in the trade-off mode to allocate the nitrogen resources under different survival strategies of either a fitness increase or a population maximum in response to the resource limitation of the environment^54^. This mechanism supports *r/K* selection in evolution and ecology^55–58^. The key element that triggered the *r/K* selection experimentally observed in the bacterial population^59–61^ was probably the nitrogen source. The trade-off mechanism of nitrogen usage in *E*. *coli* growth was highly consistent with the latest intriguing finding on the nitrogen acquisition pathways in ecosystem^62^. The osmolarity effect triggered toxicity of ammonium at high concentrations (>500 mM) was reported^37^. As the ion concentrations in the present study could reach as high as that of the ammonium toxic levels, the osmolarity effect might also contribute to the trade-off in growth strategies.

In addition, Mg^2+^ was the common decision making factor in the growth rate and the saturated density. It was reasonable that the bacterial growth required magnesium^63^, which was largely owing to its requirement in the enzymatic reactions in cells, such as translation^64,65^. However, the reason why the magnesium acted in a trade-off manner was a puzzle. We assumed that the optimal concentrations of Mg^2+^ were distinguished by the enzymes to achieve the best reaction performance in terms of either the speed or the maximum. The other assumption was that the enzymes themselves served in a trade-off manner, which was dependent on the Mg^2+^ concentration. This assumption provided a potential mechanism for magnesium flux in bacterial cells, which supported the previous reports in which the magnesium ions played a role in the speed accuracy trade-off in protein translation^66,67^.

Another intriguing finding was the secondary priority of glucose in determining the bacterial growth, indicating that the central carbohydrate metabolism was not the first prior decision step needed for growth. Considering the fact that the contributions of glucose to growth were studied intensively for decades^10,68–73^, this finding encouraged microbiologists to re-consider placing the research spotlight somewhere other than the carbon source or carbohydrate metabolism alone. Moreover, the uniform mechanism of glucose suggested that the optimization of the speed and quantity during population growth was common for carbohydrate metabolism. This optimization might benefit the conservative consumption of the carbon source for a fitness increase-associated population gain under poor nutritional conditions.

Why the trade-off was driven by NH_4_^+^ and Mg^2+^ but not by glucose remained unclear. The transporter systems responsible for the substrate uptake might play a role. The trade-off in NH_4_^+^ might be caused by the negative regulation of the transporter^74,75^. The NH_4_^+^ transporter, AmtB, is repressed in the high concentration of NH_4_^+^, which was the condition for the evaluation of the growth rate. On the contrary, the high population density might require the activated expression of AmtB to transport NH_4_^+^ at the low concentration from the environment. The regulatory mechanism agreed the finding of the high NH_4_^+^ concentration for fast growth and the low NH_4_^+^ concentration for high density. The Mg^2+^ transporters, CorA and MgtA/B, participate the Mg^2+^ uptake in substitution, depending on the concentrations of Mg^2+^ ^76^. The trade-off in Mg^2+^ might have been resulted from the difference in the regulatory mechanisms and/or the enzymatic kinetics of the two transporter systems, in response to the different concentrations of Mg^2+^. On the other hand, a wide group of membrane proteins are defined as the glucose transporters and the regulatory mechanisms of glucose uptake seemed to be sophisticated^77^. The variety of the glucose transporters possibly masked the individual working manners, in which the trader-off mechanisms might have involved. As glucose is the key source of energy for life, the uniform manner might be beneficial during the evolution.

In summary, the present study presented a successful way to apply decision tree learning to evaluate the contributions of chemical components to bacterial growth. This approach prevented bias from the investigator’s knowledge of the literature and personal experience in biology, so innovative findings on the growth decision chemicals and their working styles were obtained. Although the mechanisms remain a mystery and are purely speculation as far, the findings provide the valuable hints for the further studies on growth model. Nevertheless, the tested chemical components reached a limited number of 13, which was not large enough for a comprehensive understanding of all the chemical contributions to bacterial growth. Future study connecting the big data of the growth dynamics in living cells to varied machine learning algorithms would lead to an improved understanding of the fundamental principles of complex living systems. In addition, the *E*. *coli* strain used in the present study had a reduced genome, which might show dissimilar trait as that of the wild type strains. Nevertheless, it provided a successful example of applying the machine learning to understand the bacterial growth. The present study demonstrated that machine learning was practical for predicting and exploring the dynamics of the complex living system to acquire the new findings. That is, the trade-off mode is the top-level form of control for nitrogen distribution and the uniform approach of the secondary control for saving carbon. Machine learning applications to the life sciences is often reported in genomic studies and imaging technologies^18,23,78^. Further life science investigations would benefit from machine learning technology, by incorporating big data from the genetic information, the environmental details and the population fitness or cellular activity.

## Materials and Methods

### Bacterial strain and chemical compounds

The *E*. *coli* strain MDS42^46^ that carrying a reduced genome was used. Ten chemical compounds that are commonly used in culture media were selected for the present study. The ten compounds were *D*-glucose, ammonium sulfate, dipotassium hydrogen phosphate, potassium dihydrogen phosphate, magnesium sulfate heptahydrate, thiamine hydrochloride, iron (II) sulfate heptahydrate, sodium chloride, L-leucine and L-histidine, which were all commercially available (Wako or Sigma). The chemical compounds were dissolved in highly pure water (Direct-Q UV, Merck) at high concentrations, and then these stock solutions were sterilized using a sterile syringe filter with a 0.22 µm pore size hydrophilic PVDF membrane (Merck). The concentrations of these stock solutions were as follows: 1 M glucose, 1 M K_2_HPO_4_/KH_2_PO_4_ (5:3), 1 M MgSO_4_, 15 mM thiamine/HCl, 6.89 mM FeSO_4_, 1 M NaCl, 100 mM leucine and 100 mM histidine. The chemical combinations were prepared by mixing the stock solutions just before the experiments. A total of 243 combinations were tested during the growth assay.

### Precise and high-throughput assay on bacterial growth

To reduce the experimental errors during manipulation, the high-throughput method for precise measurements of bacterial growth was previously developed^48^ for successful applications^30,31^. Both the microplate reader and the 96-well microplate used for the measurements were precisely evaluated and carefully selected to minimize machine errors caused by the heating, shaking and sealing efficiencies before standardizing the experimental manipulation. The well-established experimental protocol with good results was described in detail elsewhere^48^. In brief, the *E*. *coli* cells were initially grown in 5 mL of M63 medium in a bioshaker (BR23-PF, Taitec) with a rotation rate of 200 rpm at 37 °C. The cell culture was stopped when the optical density (OD_600_) reached ~0.1, representing the exponential growth phase. The cell culture was subsequently stored in approximately fifty 1.5 mL microtubes (Watson) in small aliquots (100 μL per tube) for future use. These culture stocks were used only once for the growth assay, and the remainder was discarded. During the growth assay, the culture stocks were 1000-fold diluted with 5 mL of fresh media of varying chemical combinations (Table S1) in 12 mL culture tubes (Simport, Watson). The diluted cell mixtures were subsequently loaded into a 96-well microplate (Costar) in six wells of varied locations per chemical combination. The 96-well microplate was incubated in a plate reader (Epoch2, BioTek) with a rotation rate of 567 rpm at 37 °C. The temporal growth of the *E*. *coli* cells was detected at an absorbance of 600 nm, and readings were obtained at 30-min or 1-h intervals for 24 to 48 h (Tables S2, S3).

### Automated data processing for the accurate calculation of the growth parameters

The temporal OD_600_ reads representing the bacterial growth dynamics were acquired from the plate reader and exported as text files, and they were processed with Python, a programming language commonly used for data science^79^. To calculate the growth parameters, i.e., the growth rate and the saturated density, we developed a new Python program for the automated data processing of the OD_600_ reads. The program comprised the three following sections: the data tuning of the raw records, the calculation of the saturated density, and the calculation of the growth rate. Firstly, the data tuning was used to subtract the optical background of the microplate and the media from the raw records. The mean OD_600_ value of the wells filled with medium only was used as the optical background, which was subtracted from the raw OD_600_ reads of the growth curve. Secondly, the calculation of the saturated density was used to evaluate the highest optical records of the growth curve. The mean of three continuous OD_600_ reads included the maximum read, which was determined using the “argmax” in the “numpy” library. Finally, the calculation of the growth rate consisted of several steps to remove the noise and reduce the errors as follows (Fig. S1A). Step 1, extract the exponential period from the growth curve (Fig. S1B). Step 2, calculate the logarithmic slope of every two neighbouring records within the extracted period using the “gradient” in the “numpy” library. Step 3, remove the outliers from the calculated slopes in the period. Step 4, choose the maximal slope (Fig. S1B) and average it with its two neighbouring slopes. The growth rate was consequently evaluated as the mean of these three continuous slopes.

A comparison of the newly developed program with reported tools based on different platforms^80,81^ showed that the present data processing program led to the closest result for the true values acquired by manual calculation (Fig. S1C). The improvement was mostly owing to steps 1 and 3. Step 1 prevents irregular slopes outside the exponential phase to select for the calculation of the growth rate, when the noisy records, which occasionally result in a maximum logarithmic slope, occur during the lag phase of the growth curve. This step improved the accuracy of the growth rate calculation, which was demonstrated by comparing the results of the data processing with and without step 1 (Fig. S2). Step 3 deleted the false maximum of the slopes in the exponential phase from the growth calculation. Due to machine errors, irregular large records occasionally appeared during the exponential phase. It led to a false maximum in the slopes, which was considered as the outlier. To identify the outlier, the box plot, which represented the statistic distribution of the calculated slopes, was employed. The slopes outside the first quartile point were determined as the outliers and omitted from the calculation that followed. The improvement in the growth rate calculation was verified by comparing the results of the data processing with and without step 3 (Fig. S3).

### Decision tree learning with growth-chemical data sets

A non-parametric decision tree learning, classification and regression tree (CART)^45,50^ was employed. The growth parameters (i.e., the growth rate and the saturated density) and 13 chemical components were input as the target values and the target variables, respectively. Note that the logarithmic values of the chemical concentrations were used to alleviate the noise resulted from the wide parameter ranges. Decision tree learning was performed using the “DecisionTreeRegressor” package in “tree” in the scikit-learn library. Decision trees on the growth rate and saturated density were generated by a collection of rules based on the variables (chemical components) in the data set (i.e., choosing the variable for the smallest mean squared error upon regression). To evaluate the prediction precision, a five-fold cross validation was performed as follows. The growth data set was randomly divided into five groups (“cv = 5”), out of which four were used as the training data and one was used as the testing data. The mean squared errors (mse) between the predicted and the true values (*e*.*g*., growth rates) were calculated. To avoid over-fitting, the depth of the decision tree was decided according to the cross validation. Generally, deeper trees resulted in better (more precise) prediction. The depth of the decision tree was fixed when the changes in the depth did not alter the mse. The present data sets resulted in four-level decision trees for both the growth rate and the saturated density. Note that the chemical components consisting of the same compound, i.e., a cation and anion pair, occasionally had a substitutive appearance in the decision trees by chance. This result occurred because of the highly positive correlation in the changes in the concentrations of these paired chemical components, such as Na^+^ and Cl^−^. Nevertheless, these phenomena only occurred at the lower priority (branches at the third or fourth depth) of the decision trees, and thus, they did not affect the conclusions.

## Supplementary information

Supplemetary figures

Table S1

Table S2

Table S3

Table S4
