# Supplementary material for: Predicting the decision making chemicals used for bacterial growth
Source: Sci Rep. 2019 May 10;9:7251. doi: 10.1038/s41598-019-43587-8 (PMC6510730; doi:10.1038/s41598-019-43587-8)
Supplement: Supplementary file 1 — Supplemetary figures [file 41598_2019_43587_MOESM1_ESM.pdf]

Supplementary information

Predicting the decision making chemicals used for bacterial growth

Kazuha Ashino[1], Kenta Sugano[2], Toshiyuki Amagasa[2,3], Bei-Wen Ying[1,*]

[1]Graduate School of Life and Environmental Sciences, University of Tsukuba, Ibaraki 305-8572, Japan
[2]Graduate School of Systems and Information Engineering, University of Tsukuba, Ibaraki 305-8573, Japan
[3]Center for Computational Sciences, University of Tsukuba, Ibaraki 305-8577, Japan
*Correspondence: ying.beiwen.gf@u.tsukuba.ac.jp

Supplementary figures

Figures S1	p.2
Figure S2	p.3
Figure S3	p.4
Figure S4	p.5
Figure S5	p.6
Figure S6	p.7
Figure S7	p.8
Figure S8	p.9-10
Figure S9	p.11-12

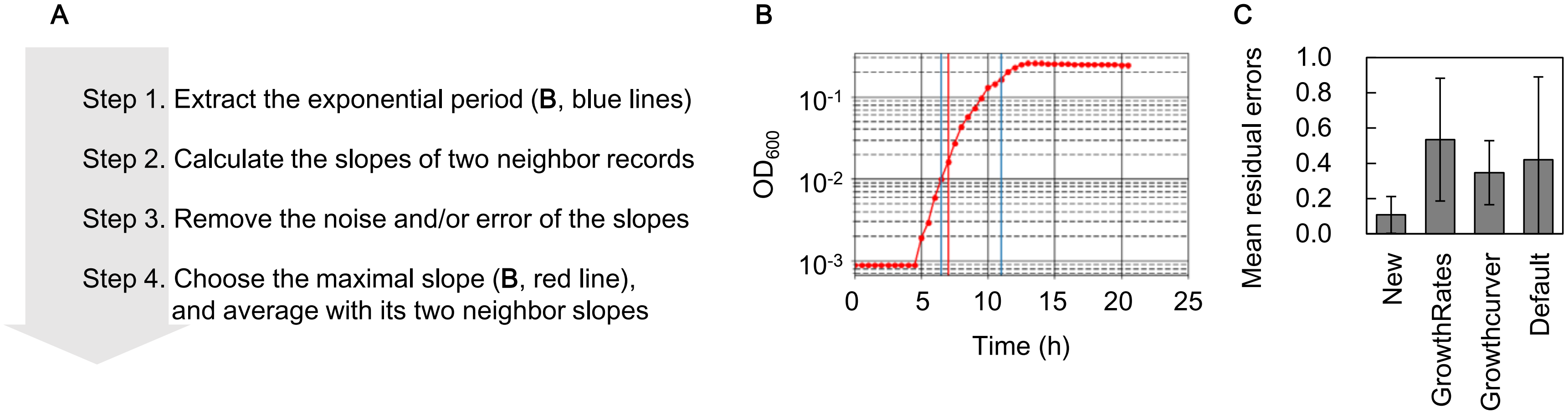

Figure S1 Improved program for calculating the growth rate with Python. A. Flowchart of data processing for calculating the growth rate. Four steps for estimating the growth rates from the growth curves are illustrated. **B.** A representative growth curve. Blue and red vertical lines represent the borders of the exponential phase and the time-point of the maximal growth rate, respectively. **C.** Comparison of varied tools for evaluating the growth rate. The mean residual errors, representing the precision of the evaluation, were calculated from 60 samples (growth curves) with four different tools. “New” and “Default” indicate the newly developed program in the present study and the tool installed on the plate reader, respectively. GrowthRate and Growthcurver are previously reported programs.

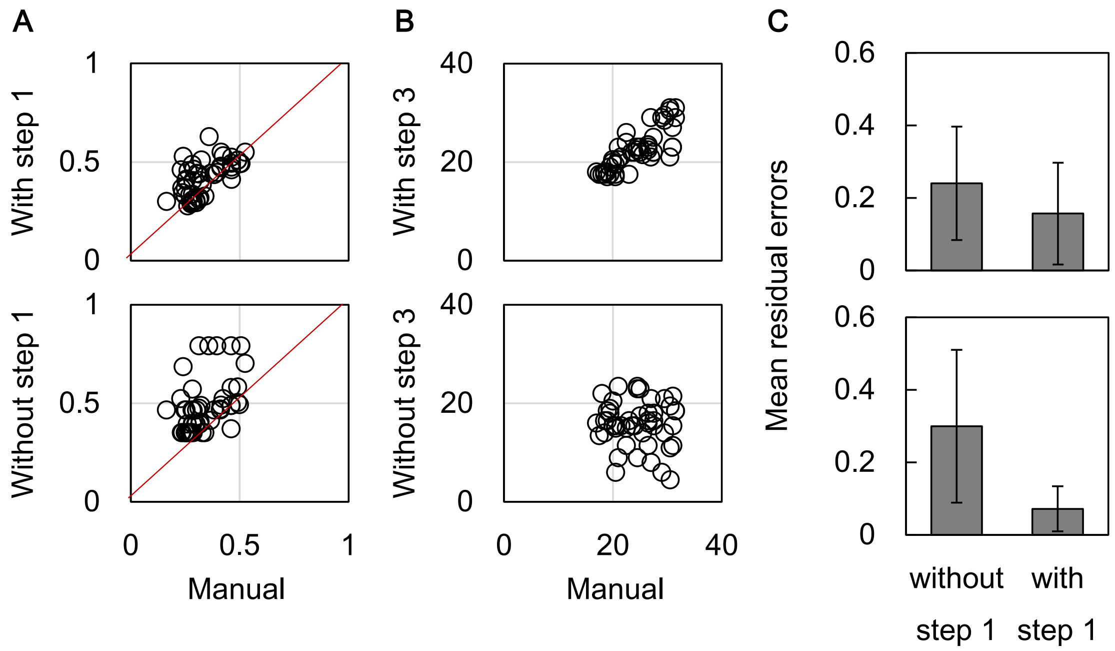

Figure S2 Improved evaluation of the growth rate due to extracting the exponential period. Whether step 1 of data processing (Fig. S1A) improved the calculation of the growth rate was examined. The growth rates calculated with or without step 1 (**A**, top and bottom panels, respectively) are plotted against the manually calculated growth rates, which were considered the true values. The red lines indicate equal values. For reference, the time points for the maximum growth rates (slopes) identified with or without step 1 (**B**, upper and bottom panels, respectively) are plotted against the manually identified time points. The mean residual errors of the growth rates and the time points (**C**, top and bottom panels, respectively) are calculated. The addition of step 1 to the program reduced the errors in the growth evaluation.

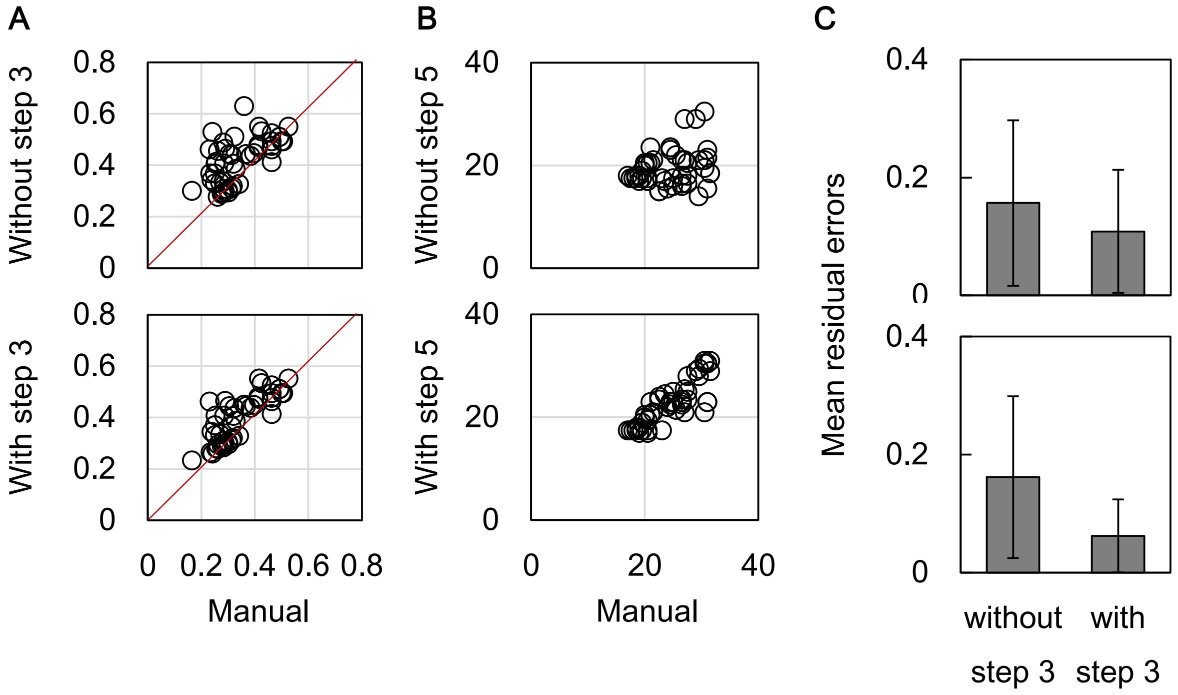

Figure S3 Improved evaluation of growth rate by removing the noise. Whether step 3 of data processing (Fig. S1A) improved the calculation of the growth rate was examined. The growth rates calculated with or without step 3 (**A**, top and bottom panels, respectively) are plotted against the manually calculated growth rates, which were considered the true values. The red lines indicate equal values. For reference, the time points of the maximum growth rates (slopes) identified with or without step 3 (**B**, upper and bottom panels, respectively) are plotted against the manually identified time points. The mean residual errors of the growth rates and the time points (**C**, upper and bottom panels, respectively) are calculated. The addition of step 3 to the program reduced the errors in the growth evaluation.

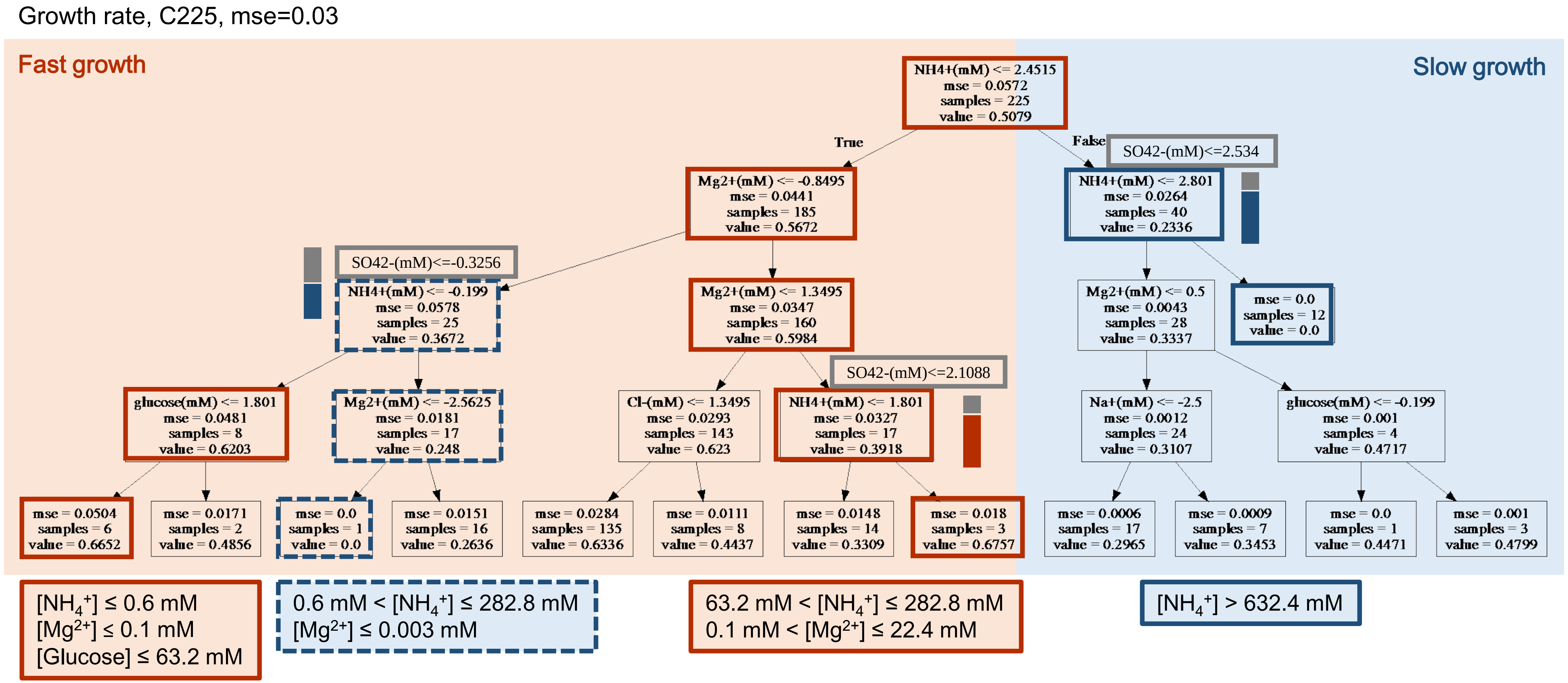

Figure S4 Decision tree for the growth rate. The data set for C225 was applied to decision tree learning to predict the growth rate. The resulting tree with a maximal depth of four is shown, and its accuracy is indicated with cross validation (mse). The chemicals predicted as decision elements in the growth rate appear in the tree. Orange and blue illustrate the branches of fast and slow growth, respectively. The name of the selected chemical and its concentration in a logarithmic scale for bifurcation, the value of cross validation for this selection, the number of data used for this selection, and the mean growth rate of the data used for this selection are summarized in the squares, from top to bottom. The squares involved in the paths of the best and worst chemical combinations for growth are highlighted with bold orange and blue lines, respectively. The grey boxes represent the alternative chemicals predicted with the decision tree. The dual-colour bars beside the boxes indicate the frequencies of the alternatives at the same levels, revealing the stability of the tree/prediction. The chemical combinations predicted to cause either fast or zero growth are summarized in the large squares, in which the ranges of the chemical concentrations are shown over linear scales.

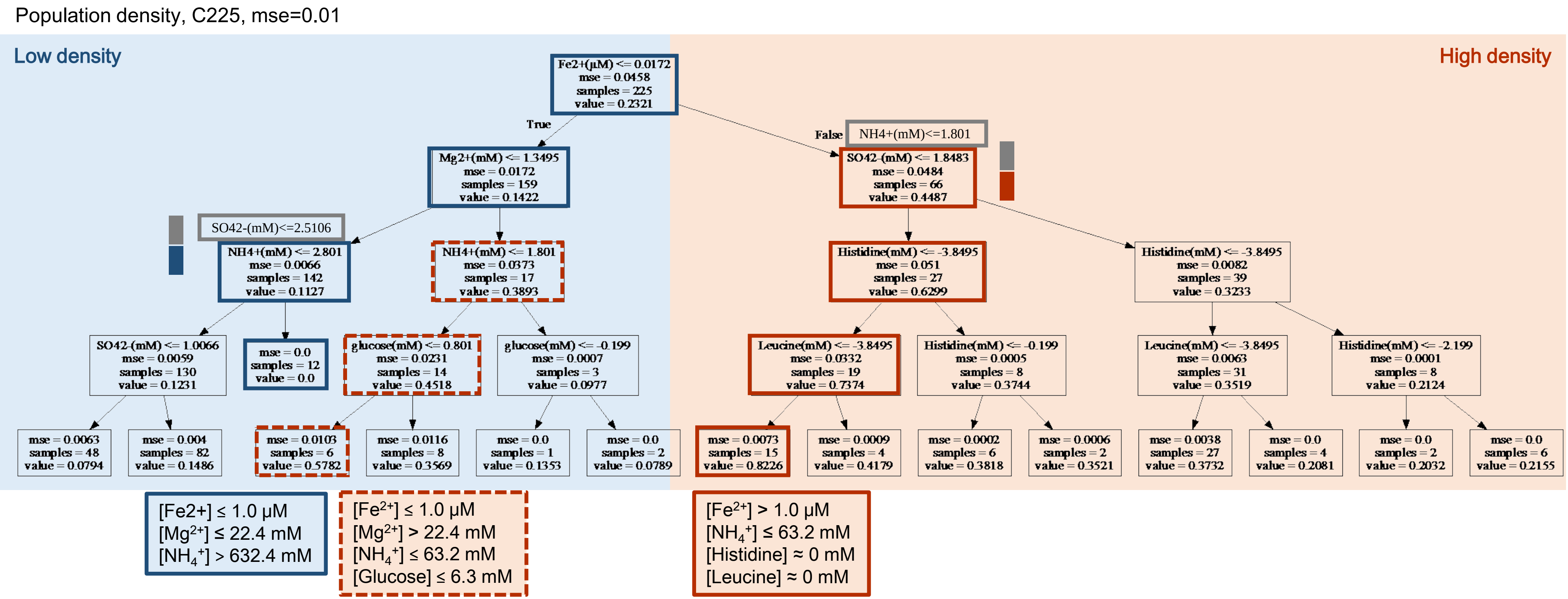

Figure S5 Decision tree of the saturated density. The C225 data set was applied to the decision tree learning to predict the saturated population density. The resulting trees with a maximal depth of four are shown, and its accuracy is indicated with cross validation (mse). The chemicals predicted to be the decision elements for the saturated density appear in the tree. Orange and blue illustrate the branches of high and low density, respectively. The name of the selected chemical and its concentration over a logarithmic scale for bifurcation, the value of cross validation of this selection, the number of data used for this selection, and the mean saturated density of the data used for this selection are summarized in the squares, from top to bottom. The squares involved in the paths of the best and worst chemical combinations for population density are highlighted with bold lines in orange and blue, respectively. The grey boxes represent the alternative chemicals predicted with the decision tree. The dual-colour bars beside the boxes indicate the frequencies of the alternatives at the same levels, revealing the stability of the tree/prediction. The chemical combinations predicted to cause either high or zero density are summarized in the large squares, for which the ranges of chemical concentrations are shown in the linear scales.

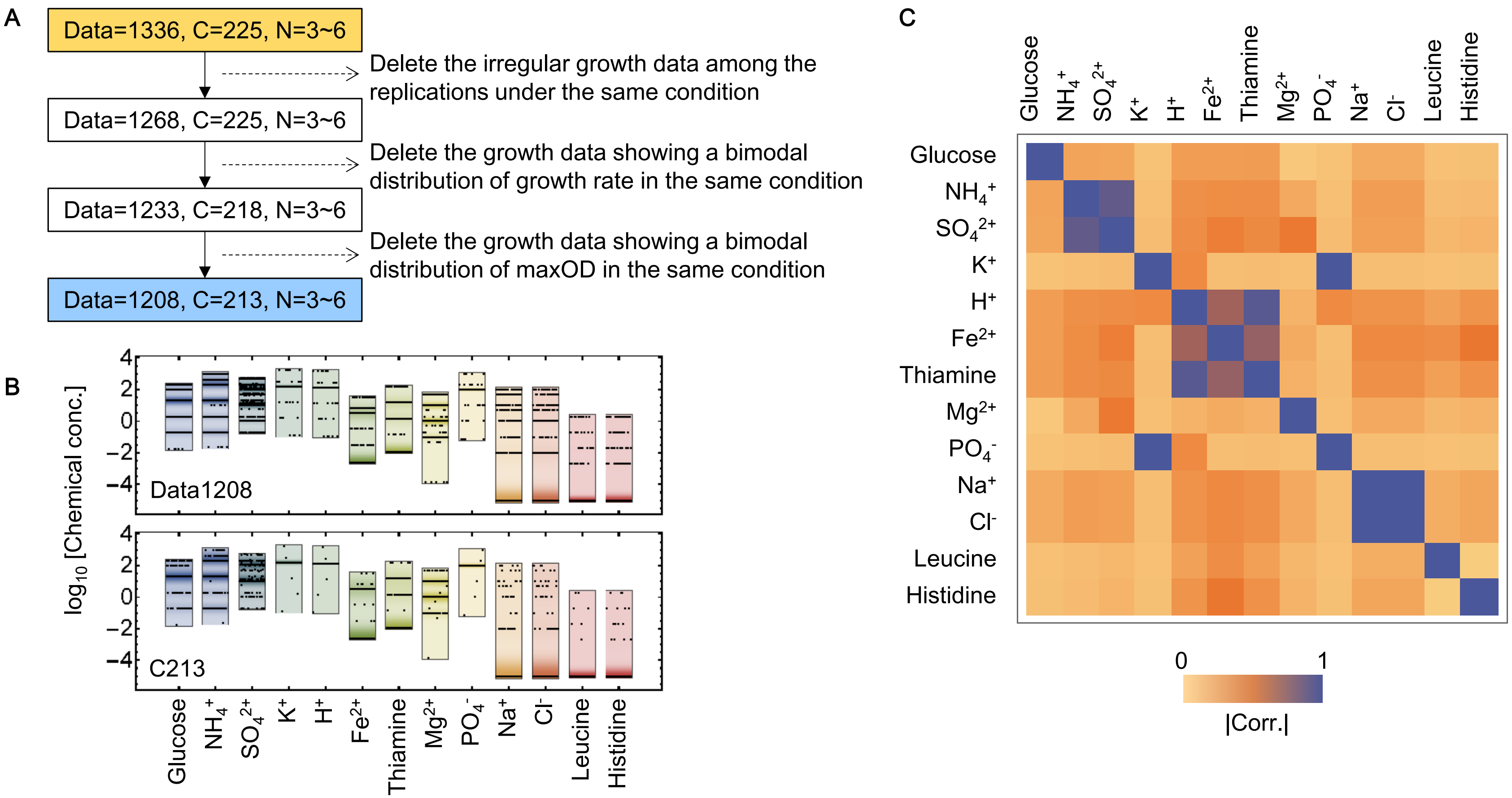

Figure S6 Data distribution and the chemical combinations of the processed data sets. A. Flowchart of the data refinement. Data, C, and N represent the numbers of the individual growth curves acquired in the assay, the tested combinations from the ten chemical compounds, and the biological replicates per combination, respectively. The data set highlighted in orange was the purified data set used in the following analyses. **B.** Data distributions at the single chemical level. A total of 13 chemicals, which are encompassed within the ten compounds, are indicated. The data distributions at individual chemical levels are shown as coloured bars. The tested concentrations of these 13 chemicals are spotted in black, within the corresponding distribution bars. Those condensed spots appear as black lines. The top and bottom panels indicate the distributions of 1208 individual growth curves and the 213 combinations, respectively. **C.** Relationships between the concentration changes of 13 individual chemicals. The matrix represents the correlations of the changes in the concentrations of any two chemicals. The gradation from light orange to dark blue indicates the correlation coefficients from low to high.

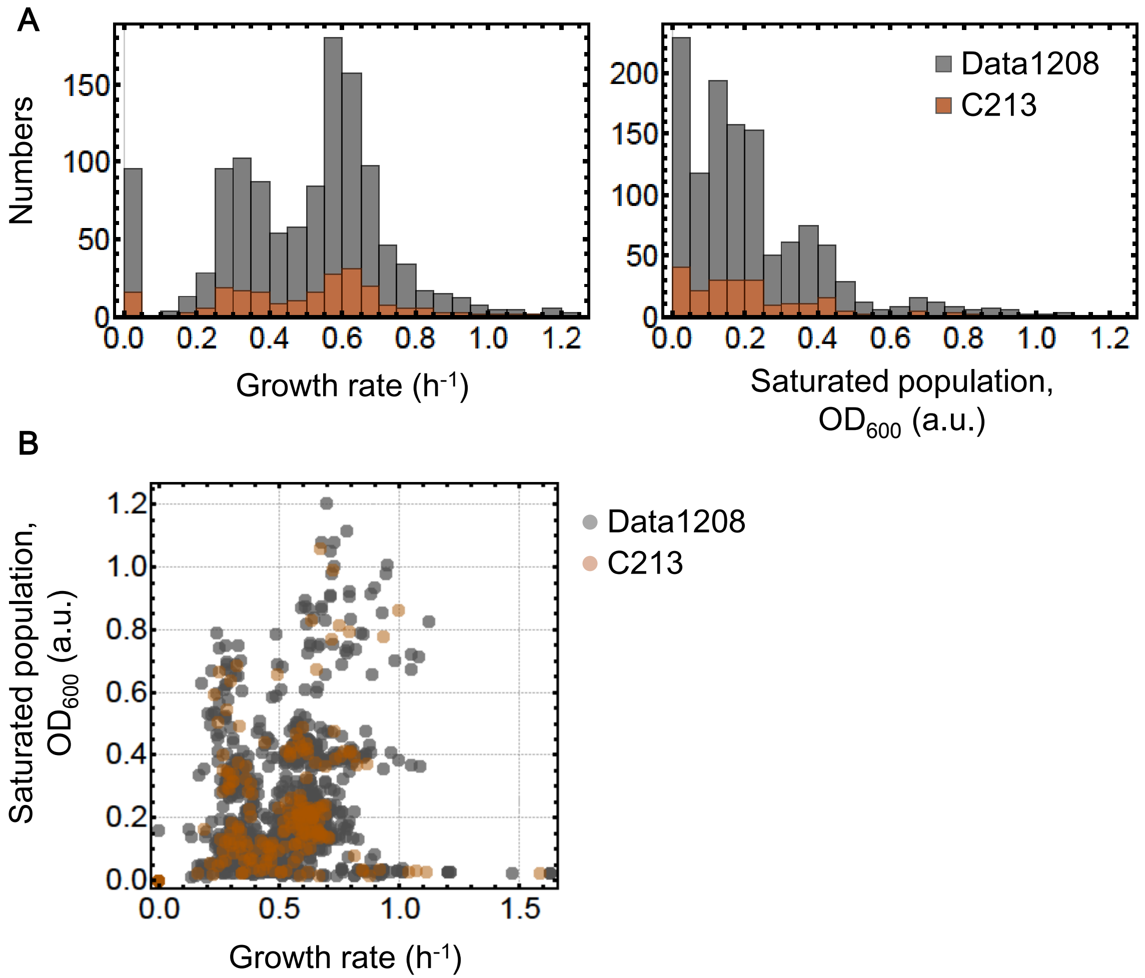

Figure S7 Growth rates and saturated population densities of the processed data sets. A. Histograms of the growth rate and the saturated density. The left and right panels show the histograms of the growth rate and the saturated density, respectively. **B.** Relation between the growth rate and the saturated density. The Spearman rank correlation coefficients of Data1208 and C213 are 0.33 (p=5e-33) and 0.61 (p=7e-23), respectively. The Data1208 and C213 data sets are indicated in grey and bronze, respectively.

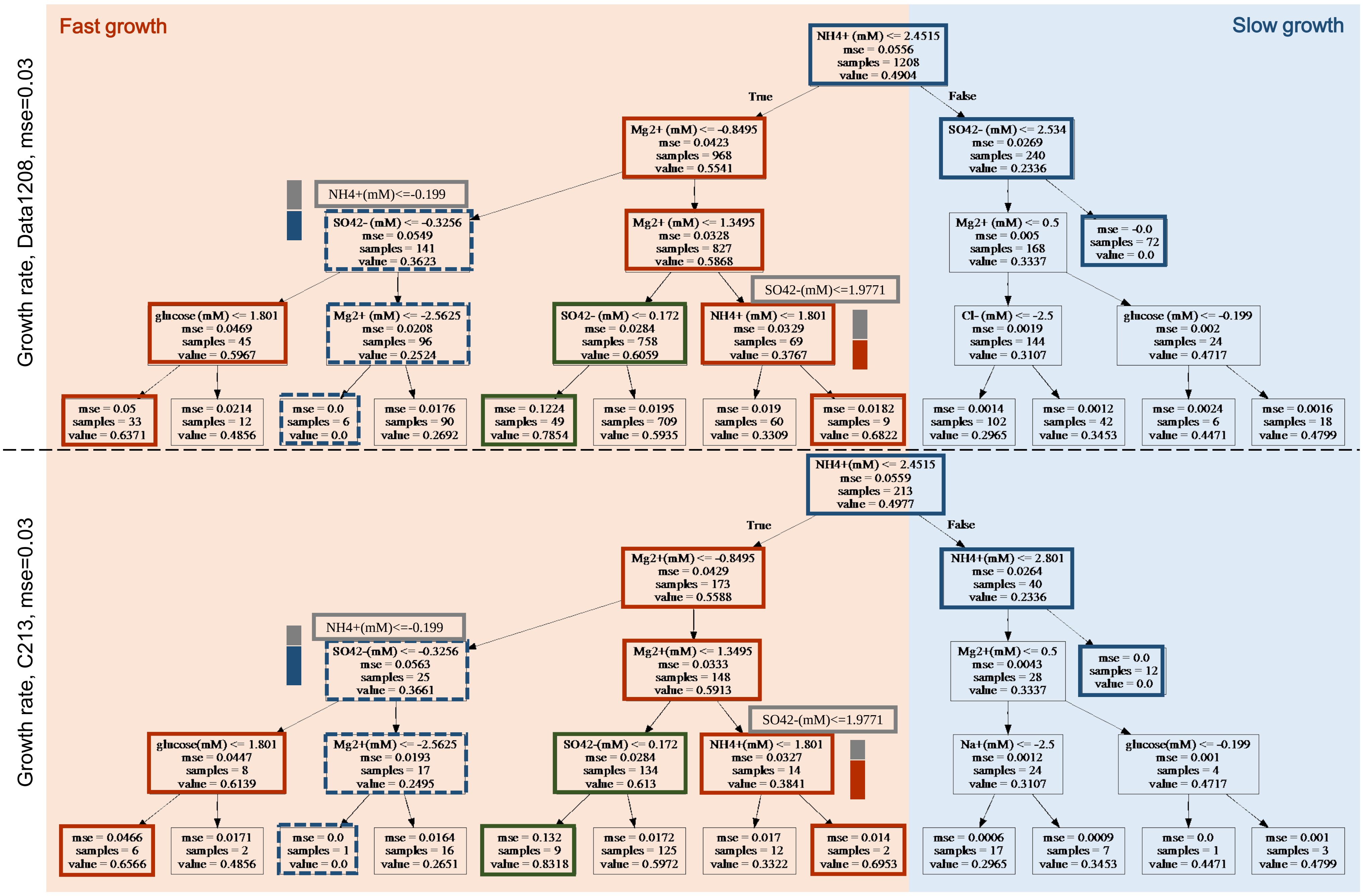

Fast growth
Slow growth
Growth rate, Data1208, mse=0.03
NH4+ (mM) <= 2.4515 mse = 0.0556 samples = 1208 value = 0.4904
True
False
Mg2+ (mM) <= -0.8495 mse = 0.0423 samples = 968 value = 0.5541
SO42- (mM) <= 2.534 mse = 0.0269 samples = 240 value = 0.2336
NH4+(mM)<=-0.199
SO42- (mM) <= -0.3256 mse = 0.0549 samples = 141 value = 0.3623
Mg2+ (mM) <= 1.3495 mse = 0.0328 samples = 827 value = 0.5868
Mg2+ (mM) <= 0.5 mse = 0.005 samples = 168 value = 0.3337
mse = -0.0 samples = 72 value = 0.0
SO42-(mM)<=1.9771
glucose (mM) <= 1.801 mse = 0.0469 samples = 45 value = 0.5967
Mg2+ (mM) <= -2.5625 mse = 0.0208 samples = 96 value = 0.2524
SO42- (mM) <= 0.172 mse = 0.0284 samples = 758 value = 0.6059
NH4+ (mM) <= 1.801 mse = 0.0329 samples = 69 value = 0.3767
Cl- (mM) <= -2.5 mse = 0.0019 samples = 144 value = 0.3107
glucose (mM) <= -0.199 mse = 0.002 samples = 24 value = 0.4717
mse = 0.05 samples = 33 value = 0.6371
mse = 0.0214 samples = 12 value = 0.4856
mse = 0.0 samples = 6 value = 0.0
mse = 0.0176 samples = 90 value = 0.2692
mse = 0.1224 samples = 49 value = 0.7854
mse = 0.0195 samples = 709 value = 0.5935
mse = 0.019 samples = 60 value = 0.3309
mse = 0.0182 samples = 9 value = 0.6822
mse = 0.0014 samples = 102 value = 0.2965
mse = 0.0012 samples = 42 value = 0.3453
mse = 0.0024 samples = 6 value = 0.4471
mse = 0.0016 samples = 18 value = 0.4799
Growth rate, C213, mse=0.03
NH4+(mM) <= 2.4515 mse = 0.0559 samples = 213 value = 0.4977
True
False
Mg2+(mM) <= -0.8495 mse = 0.0429 samples = 173 value = 0.5588
NH4+(mM) <= 2.801 mse = 0.0264 samples = 40 value = 0.2336
NH4+(mM)<=-0.199
SO42-(mM) <= -0.3256 mse = 0.0563 samples = 25 value = 0.3661
Mg2+(mM) <= 1.3495 mse = 0.0333 samples = 148 value = 0.5913
Mg2+(mM) <= 0.5 mse = 0.0043 samples = 28 value = 0.3337
mse = 0.0 samples = 12 value = 0.0
SO42-(mM)<=1.9771
glucose(mM) <= 1.801 mse = 0.0447 samples = 8 value = 0.6139
Mg2+(mM) <= -2.5625 mse = 0.0193 samples = 17 value = 0.2495
SO42-(mM) <= 0.172 mse = 0.0284 samples = 134 value = 0.613
NH4+(mM) <= 1.801 mse = 0.0327 samples = 14 value = 0.3841
Na+(mM) <= -2.5 mse = 0.0012 samples = 24 value = 0.3107
glucose(mM) <= -0.199 mse = 0.001 samples = 4 value = 0.4717
mse = 0.0466 samples = 6 value = 0.6566
mse = 0.0171 samples = 2 value = 0.4856
mse = 0.0 samples = 1 value = 0.0
mse = 0.0164 samples = 16 value = 0.2651
mse = 0.132 samples = 9 value = 0.8318
mse = 0.0172 samples = 125 value = 0.5972
mse = 0.017 samples = 12 value = 0.3322
mse = 0.014 samples = 2 value = 0.6953
mse = 0.0006 samples = 17 value = 0.2965
mse = 0.0009 samples = 7 value = 0.3453
mse = 0.0 samples = 1 value = 0.4471
mse = 0.001 samples = 3 value = 0.4799

Figure S8 Decision tree of the growth rate predicted using the clean data sets. The purified Data1208 and C213 data sets (upper and bottom panels, respectively) were applied to the decision tree learning to predict the growth rate. The resulting trees with a maximal depth of four are shown, and the accuracy is indicated with cross validation (mse). The chemicals predicted to be the decision elements for the growth rate appear in the tree. Orange and blue illustrate the branches of fast and slow growth, respectively. The name of the selected chemical and its concentration over a logarithmic scale for bifurcation, the value of cross validation of this selection, the number of data used for this selection, and the mean growth rate of the data used for this selection are summarized in the squares, from top to bottom. The squares involved in the paths directed towards fast growth are highlighted with bold lines in orange and green, and those with zero growth are in blue. The grey boxes represent the alternative chemicals predicted with the decision tree. The dual-colour bars beside the boxes indicate the frequencies of the alternatives at the same levels, revealing the stability of the tree/prediction. The decision trees were highly identical to those acquired with the noisy data sets (Data1336, C225). An alternative path for fast growth (highlighted in green) was newly determined.

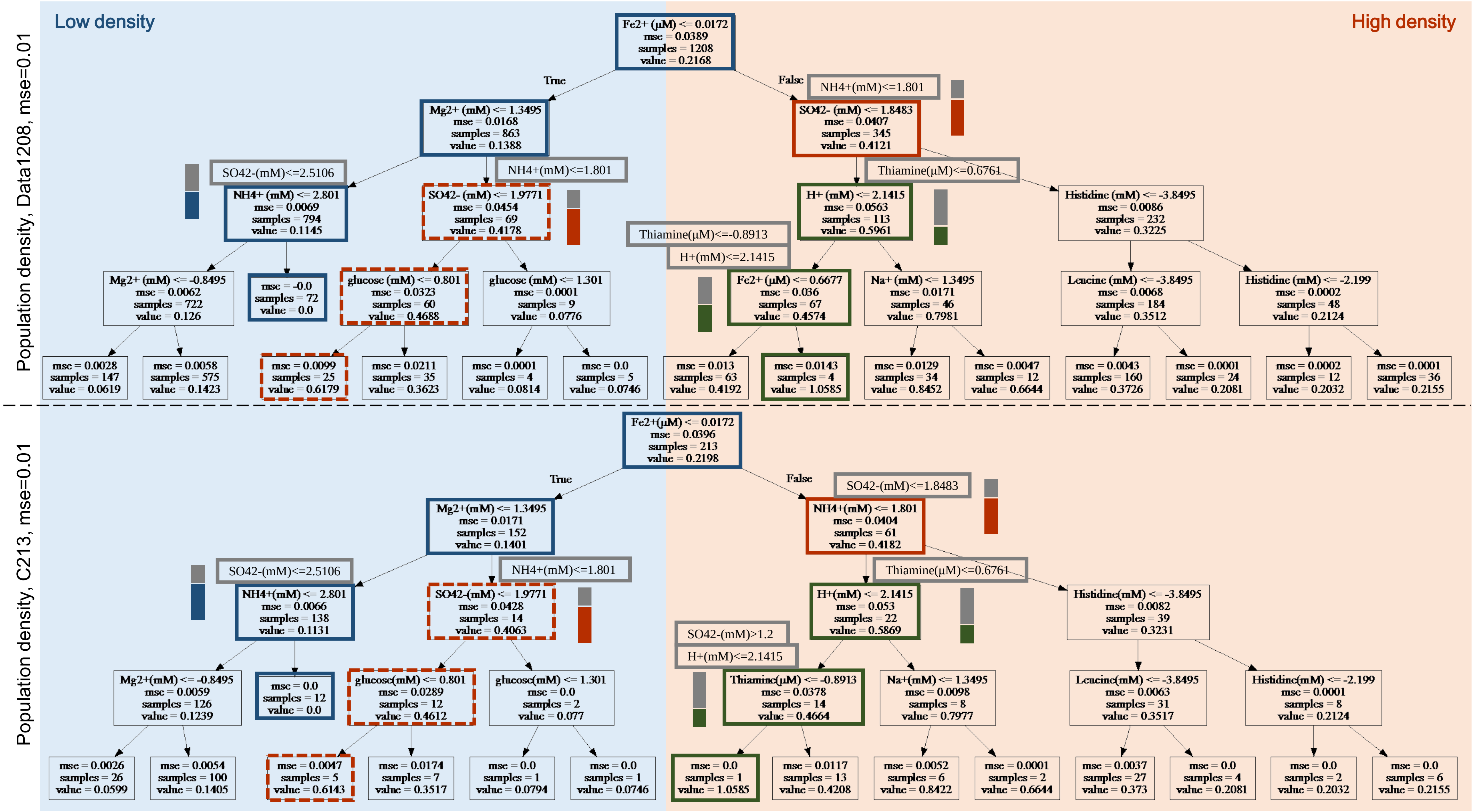

Low density
High density
Population density, Data1208, mse=0.01
Fe2+ (μM) <= 0.0172
mse = 0.0389
samples = 1208
value = 0.2168
True
False
Mg2+ (mM) <= 1.3495
mse = 0.0168
samples = 863
value = 0.1388
NH4+(mM)<=1.801
SO42- (mM) <= 1.8483
mse = 0.0407
samples = 345
value = 0.4121
SO42-(mM)<=2.5106
NH4+(mM)<=1.801
Thiamine(μM)<=0.6761
NH4+ (mM) <= 2.801
mse = 0.0069
samples = 794
value = 0.1145
SO42- (mM) <= 1.9771
mse = 0.0454
samples = 69
value = 0.4178
H+ (mM) <= 2.1415
mse = 0.0563
samples = 113
value = 0.5961
Histidine (mM) <= -3.8495
mse = 0.0086
samples = 232
value = 0.3225
Thiamine(μM)<=-0.8913
H+(mM)<=2.1415
Mg2+ (mM) <= -0.8495
mse = 0.0062
samples = 722
value = 0.126
mse = -0.0
samples = 72
value = 0.0
glucose (mM) <= 0.801
mse = 0.0323
samples = 60
value = 0.4688
glucose (mM) <= 1.301
mse = 0.0001
samples = 9
value = 0.0776
Fe2+ (μM) <= 0.6677
mse = 0.036
samples = 67
value = 0.4574
Na+ (mM) <= 1.3495
mse = 0.0171
samples = 46
value = 0.7981
Leucine (mM) <= -3.8495
mse = 0.0068
samples = 184
value = 0.3512
Histidine (mM) <= -2.199
mse = 0.0002
samples = 48
value = 0.2124
mse = 0.0028
samples = 147
value = 0.0619
mse = 0.0058
samples = 575
value = 0.1423
mse = 0.0099
samples = 25
value = 0.6179
mse = 0.0211
samples = 35
value = 0.3623
mse = 0.0001
samples = 4
value = 0.0814
mse = 0.0
samples = 5
value = 0.0746
mse = 0.013
samples = 63
value = 0.4192
mse = 0.0143
samples = 4
value = 1.0585
mse = 0.0129
samples = 34
value = 0.8452
mse = 0.0047
samples = 12
value = 0.6644
mse = 0.0043
samples = 160
value = 0.3726
mse = 0.0001
samples = 24
value = 0.2081
mse = 0.0002
samples = 12
value = 0.2032
mse = 0.0001
samples = 36
value = 0.2155
Population density, C213, mse=0.01
Fe2+(μM) <= 0.0172
mse = 0.0396
samples = 213
value = 0.2198
True
False
Mg2+(mM) <= 1.3495
mse = 0.0171
samples = 152
value = 0.1401
SO42-(mM)<=1.8483
NH4+(mM) <= 1.801
mse = 0.0404
samples = 61
value = 0.4182
SO42-(mM)<=2.5106
NH4+(mM)<=1.801
Thiamine(μM)<=0.6761
NH4+(mM) <= 2.801
mse = 0.0066
samples = 138
value = 0.1131
SO42-(mM) <= 1.9771
mse = 0.0428
samples = 14
value = 0.4063
H+(mM) <= 2.1415
mse = 0.053
samples = 22
value = 0.5869
Histidine(mM) <= -3.8495
mse = 0.0082
samples = 39
value = 0.3231
SO42-(mM)>1.2
H+(mM)<=2.1415
Mg2+(mM) <= -0.8495
mse = 0.0059
samples = 126
value = 0.1239
mse = 0.0
samples = 12
value = 0.0
glucose(mM) <= 0.801
mse = 0.0289
samples = 12
value = 0.4612
glucose(mM) <= 1.301
mse = 0.0
samples = 2
value = 0.077
Thiamine(μM) <= -0.8913
mse = 0.0378
samples = 14
value = 0.4664
Na+(mM) <= 1.3495
mse = 0.0098
samples = 8
value = 0.7977
Leucine(mM) <= -3.8495
mse = 0.0063
samples = 31
value = 0.3517
Histidine(mM) <= -2.199
mse = 0.0001
samples = 8
value = 0.2124
mse = 0.0026
samples = 26
value = 0.0599
mse = 0.0054
samples = 100
value = 0.1405
mse = 0.0047
samples = 5
value = 0.6143
mse = 0.0174
samples = 7
value = 0.3517
mse = 0.0
samples = 1
value = 0.0794
mse = 0.0
samples = 1
value = 0.0746
mse = 0.0
samples = 1
value = 1.0585
mse = 0.0117
samples = 13
value = 0.4208
mse = 0.0052
samples = 6
value = 0.8422
mse = 0.0001
samples = 2
value = 0.6644
mse = 0.0037
samples = 27
value = 0.373
mse = 0.0
samples = 4
value = 0.2081
mse = 0.0
samples = 2
value = 0.2032
mse = 0.0
samples = 6
value = 0.2155

Figure S9 Decision tree of the saturated density predicted with the clean data sets. The purified Data1208 and C213 data sets (the top and bottom panels, respectively) were applied to the decision tree learning to predict the saturated population density. The resulting trees with a maximal depth of four are shown, and the accuracy is indicated with cross validation (mse). The chemicals predicted to be the decision elements for saturated density appear in the tree. Orange and blue illustrate the branches of high and low density, respectively. The name of the selected chemical and its concentration in a logarithmic scale for bifurcation, the value of cross validation for this selection, the number of data used for this selection, and the mean saturated density of the data used for this selection are summarized in the squares, from top to bottom. The squares involved in the paths directed towards the high density are highlighted with bold lines in orange and green, and those moving towards zero density are in blue. The grey boxes represent the alternative chemicals predicted with the decision tree. The dual-colour bars beside the boxes indicate the frequencies of the alternatives at the same levels, revealing the stability of the tree/prediction. Low-density branches were highly identical to those based on the noisy data sets (Data1336, C225). An alternative path for the highest density was newly determined (highlighted in green).
